# Eculizumab-Related Abortion in a Woman with Paroxysmal Nocturnal Hemoglobinuria: A Case Report

**Published:** 2019

**Authors:** Adrián Rodríguez-Ferreras, Lucia Velasco-Roces

**Affiliations:** - Pharmacy Department, Hospital Universitario Central de Asturias, Oviedo, Spain

**Keywords:** Abortion, Complement activation, Eculizumab, Miscarriage, Pregnancy

## Abstract

**Background:**

The use of eculizumab during pregnancy has generally been discouraged. Published data on related studies provides conflicting information and establishing a benefit-risk relationship proves to be a complicated task. Miscarriage rates, concomitant medications, and the stages of pregnancy when eculizumab treatment was initiated varied among the patients included in the case series. The aim of this report is to discuss eculizumab use during pregnancy.

**Case Presentation:**

A case of a woman diagnosed with Paroxysmal Nocturnal Hemoglobinuria (PNH) and treated with eculizumab, who expressed desire for pregnancy is presented. Six months after her eculizumab treatment, the patient experienced spontaneous abortion in her first trimester. The direct relation between eculizumab and the miscarriage is not clear. Other factors may have influenced this case, thus demonstrating the difficulty of managing pregnancy in women with PNH.

**Conclusion:**

Controversy on eculizumab risk during pregnancy encourages further review on its use, highlighting the importance to assess each case individually.

## Introduction

PNH is a clonal blood disorder which leads to a higher risk for increased venous and arterial thrombosis. It can present with hemolytic-anemia, smooth muscle dystonia, thrombosis, and bone marrow failure (1). There is a clear benefit of eculizumab treatment in symptomatic patients, both in reduction of biochemical markers of hemolysis and in associated symptoms, especially fatigue (2).

Intravascular hemolysis and anemia are frequently more severe during pregnancy, with greater transfusion requirements (3). Maternal and fetal morbi-mortality is higher among pregnant women with PNH, and the risks are even higher during the postpartum period. Fetal mortality has been reported to be between 4% and 9% and maternal mortality between 8% and 20.8%, mainly related to postpartum thrombosis (4). Some authors recommend prophylaxis against thromboembolic events during pregnancy and for 6 weeks postpartum (5), but there are no clearly established criteria and it is an issue of active debate (6).

The fact that eculizumab crosses the placenta in sufficient quantity to cause fetal harm is also controversial. One report concluded that eculizumab treatment during pregnancy does not impair the complement function in the newborn (7). A recent publication suggests that eculizumab crosses the placenta at low levels (detected in 7 of 20 cord-blood samples) being insufficient to affect complement activation (4). On the other hand, it has been proved that the four human IgG subclasses are all actively transported across the placenta (7,8) and contraception in women with PNH has been recommended due to eculizumab's potential for teratogenicity (1, 8).

Pregnancy has generally been discouraged in women with PNH (9). Eculizumab treatment improves quality of life of young women and they feel symptomatically better. The question of eculizumab use during pregnancy is increasingly raised and managing these patients presents a major challenge (4, 11).

## Case Presentation

The case of a nulliparous 38-year-old female with PNH, diagnosed in August 2016, with a large granulocyte clone size (80.36%) is presented. The PNH test also showed the following subtypes of red cells; type-III 7.81%, type-II 20.27%, and type-I 71.92%. She had frequent esophageal spasms, without smooth muscle dystonia, asthenia that results in certain limitation of some daily activities and self-limited chest pain. Additionally, she had a biological data of high disease activity (Persistently high LDH, increased D-dimer and high ratio between type-II/III red cells). She never required blood transfusion or symptomatic treatment, and her hemoglobin values were always within the normal range (13 *g/dl* at diagnosis; never below 12.4 *g/dl*).

## Results

A year after diagnosis, conceding to the patient's desire for pregnancy and the potential increased risk, eculizumab treatment was proposed despite no signs of hemolysis. Initially, LDH and D-dimer values were 658 *U/L* and 821 *ng/ml*, respectively. An MRI was performed to evaluate ferric overload and occult thrombosis, with normal imaging test. Prior to starting treatment, antibiotic prophylaxis was given for subsequent vaccination against meningococcus (6). In August 2017, eculizumab treatment was administered following the regular regimen of 600 *mg* per week for the first 4 weeks and 900 *mg* every 14 days thereafter. No antithrombotic prophylaxis was performed. Once induction phase was completed, a clear clinical improvement was observed, disappearance of esophageal spasm episodes, asthenia, and chest pain. Tolerance was good, with only a mild headache 24 *hr* post-infusion. LDH and D-dimer values returned to normal.

In January 2018, six months after her eculizumab treatment, the patient came to the emergency department of our hospital due to heavy vaginal bleeding and abdominal pain (a total of 16 eculizumab doses received until that moment). She was at 6+4 weeks of amenorrhea. Her physical examination revealed normal genitalia with vaginal remains after Valsalva; these were sent for pathological anatomy analysis. In her ultrasound scan, a 17 *mm* endometrium with two anechoic images of 2 and 4 *mm*, respectively, was observed (with normal left and right annexes). The value of βHCG was 910.5 *IU/L*. The initial diagnosis was either early/ectopic pregnancy or miscarriage. Given these findings, hormonal monitoring was decided; two days and one week later, βHCG values were 409.9 *IU/L* and 13.1 *IU/L*, respectively. Hormone level changes and sample results confirmed the diagnosis of miscarriage in the first trimester. Menstruation after the miscarriage was heavy, with presyncopal episode.

At present, the patient continues eculizumab treatment and is hematologically stable, without clinical symptoms or hemolysis. She still expresses desire for gestation, despite knowing the risks involved.

## Discussion

According to the consensus guidelines for the diagnosis and treatment of PNH by the Spanish Society of Hematology, patients with chronic intravascular hemolytic anemia, LDH values that are 1.5 times above the normal upper limit, and clinical symptoms due to hemolytic anemia, regardless of transfusion requirements are considered candidates for eculizumab treatment. Smooth muscle dystonia, heart failure, or pulmonary hypertension are also indications for treatment. Additionally, due to the high thrombotic risk in pregnant women with PNH, and the limited evidence of eculizumab use during pregnancy, its use must be assessed individually (10). In our case, these criteria were met and after multidisciplinary assessment, eculizumab treatment was approved. However, some might consider this patient not a candidate due to her hematological profile and the fact that she did not require transfusion support meant a relatively non-severe limitation of daily life activities.

Prophylactic low molecular weight heparin is often prescribed to pregnant women, as long as there is no contraindication. It is usually initiated during the third trimester and continued for 6–12 weeks postpartum. Many recommend it when clone size is more than 50% (12). Previous studies confirm an odds ratio of 1.64 of thrombosis for every 10% expansion of the clone size (13). Our patient had a clone of 80.36% and was in the first trimester, so according to this, the suitability for anticoagulation in this case remains unknown.

Spontaneous abortion rate in women without PNH during the first trimester is around 17% (4). Case series have shown evidence improving this rate as well as maternal complications during pregnancy (11, 12). However, pregnant women treated with eculizumab are not risk-free and the relationship between eculizumab and cases of spontaneous abortion is not clearly established. Eculizumab is considered class C medication. Animal studies showed a rare complication of retinal dysplasia and umbilical herniation (1, 8). A recent publication assessed the safety and efficacy of eculizumab in 61 pregnant patients with PNH. Six spontaneous abortions occurred during the first trimester and two stillbirths occurred in one patient, at 30 and 32 weeks of gestation (total rate of miscarriage of 8%) (4). In this study, the median age at the start of their pregnancies was 29 years (range: 18 to 40). In another review that included 14 patients, wherein median age was 30.2 years (range: 22 to 37), all patients successfully gave birth (14). Cases of spontaneous abortions have been reported in other case series, with varying ages among pregnant women (13). The stage of pregnancy when eculizumab was started and its treatment duration varied (15). Therefore, it is not possible to establish a recommendation on the optimal timing and duration of eculizumab treatment during pregnancy.

Our patient suffered a spontaneous abortion in her first trimester, six months after receiving eculizumab. The cause of miscarriage could be multi-factorial. The patient’s age was above the median of published cases, which means an added difficulty in carrying a full-term pregnancy. According to the clone size, as well as in previously reported cases, anticoagulant therapy could have been initiated. The optimal time to start eculizumab treatment during pregnancy is unclear and the influence it may have is largely inconclusive. The need to evaluate the benefit-risk relationship in each patient becomes essential in determining the need for treatment, especially for a patient who exhibited limited symptoms, such as our case.

## Conclusion

The safety evidence of eculizumab during pregnancy remains unclear. The drug's technical Data Sheet and some publications warn about its potential teratogenic risk and discourage its use. On the other hand, case series and recent reviews, a few published in high-impact journals, support it. Establishing specific usage recommendations in this group of patients proves to be complicated, due to the limitation of conducting studies given the low occurrence of pregnant women with PNH and ethical concerns.
